# Safety and Effectiveness of Colonic Stenting for Ileocecal Valve Obstruction and Usefulness of Two-Step Strategy: Single-Center Retrospective Study

**DOI:** 10.3390/jcm14030826

**Published:** 2025-01-27

**Authors:** Gota Fujisawa, Rei Ishibashi, Shuntaro Yoshida, Ryo Kondo, Masahiro Hata, Yukiko Oya, Nariaki Odawara, Ayako Nakada, Yumiko Komine, Ryunosuke Hakuta, Naminatsu Takahara, Nobumi Suzuki, Yousuke Nakai, Hiroyuki Isayama, Mitsuhiro Fujishiro

**Affiliations:** 1 Department of Gastroenterology, Graduate School of Medicine, The University of Tokyo, Tokyo 113-8655, Japan; 2 Department of Internal Medicine, Institute of Gastroenterology, Tokyo Women’s Medical University, Tokyo 162-8666, Japan; 3 Department of Gastroenterology, Juntendo University Graduate School of Medicine, Tokyo 113-8431, Japan

**Keywords:** self-expandable metallic stent, malignant large bowel obstruction, ileocecal obstruction

## Abstract

**Background:** Endoscopic self-expandable metallic stent (SEMS) placement is a widely accepted treatment for malignant left-sided colorectal obstruction (LSO) because of its lower invasiveness and quicker symptomatic relief compared to surgery. However, SEMS placement for ileocecal valve obstruction (ICVO) has not been established due to its technical difficulties. **Methods:** This single-center retrospective study compared the clinical outcomes of patients who underwent SEMS placement for ICVO (ICVO group, *n* = 13) and LSO (LSO group, *n* = 146). Particularly in cases with severe small-intestine dilation, we applied a “Two-Step Strategy”, which involved long intestinal tube insertion followed by SEMS placement to ensure safety and overcome technical challenges. **Results:** Patients in the ICVO group were significantly more likely to undergo SEMS placement with the Two-Step Strategy compared to those in the LSO group (76.9% vs. 6.9%, *p* < 0.001). Both groups achieved similarly high technical and clinical success rates (100% vs. 98.6%, *p* = 1.000; 92.3% vs. 88.4%, *p* = 1.000), and the incidence of adverse events also showed no significant difference between the groups (7.7% vs. 13.0%; *p* = 1.000). Furthermore, the median time to recurrent colorectal obstruction and survival time after SEMS placement did not differ between patients with palliative stenting for ICVO and LSO (not reached vs. 430 days, *p* = 0.586; 119 days vs. 200 days, *p* = 0.303). **Conclusions**: SEMS placement for malignant ICVO is as safe and effective as it is for malignant LSO, and the Two-Step Strategy might be useful in ICVO cases.

## 1. Introduction

Endoscopic self-expandable metallic stent (SEMS) placement is a widely accepted, minimally invasive procedure for the management of malignant colorectal obstruction in cases of potentially curative and unresectable disease [1,2,3,4]. Initially, endoscopic SEMS placement was mainly used to treat left-sided colorectal obstruction (LSO) [5,6], and surgical ileostomy was preferred for right-sided colonic obstruction. In recent years, due to increasing evidence regarding the safety and effectiveness of SEMS placement, its application has been expanded to right-sided colonic obstruction [7,8]. However, little is known regarding its usage for ileocecal valve obstruction (ICVO) [9,10,11]. This is mainly because SEMS placement is technically demanding for ICVO due to anatomical reasons, such as the long distance from the anus and the original angulated anatomy [10]. Hence, technical adjustments are necessary to apply endoscopic SEMS placement for ICVO.

In most malignant colorectal obstructions, the primary pathophysiology is large-bowel dilation, whereas in ICVO, it is small-intestine dilation. Therefore, in cases of ICVO, the placement of a long intestinal tube is expected to provide the effective decompression of the small intestine and ensure safety during subsequent colonic stenting. In addition, we could overcome the technical difficulties of stenting by injecting contrast media or performing antegrade manipulation through the long intestinal tube at the time of stenting. For these reasons, particularly in cases with severe small-intestine dilation, our institution has adopted a stepwise strategy for ICVO, in which an ileus tube is placed first, and the SEMS is inserted after small-intestine decompression and the advance of the intestinal tube, which we name the “Two-Step Strategy”.

In this study, we aimed to compare the outcomes of SEMS placement, the most established procedure in colorectal stenting, for ICVO with those for LSO and report the usefulness of the Two-Step Strategy for SEMS placement for malignant ICVO.

## 2. Materials and Methods

### 2.1. Study Design

This retrospective study was conducted at The University of Tokyo Hospital to investigate the safety and effectiveness of endoscopic SEMS placement for malignant colorectal obstructions. Written informed consent for endoscopic intervention was obtained from all patients before the procedure. This study was approved by the ethics committee of the hospital and consent for the use of data for research was obtained on an opt-out basis (Institutional Review Board number: 2058).

### 2.2. Patients

**Figure 1:**
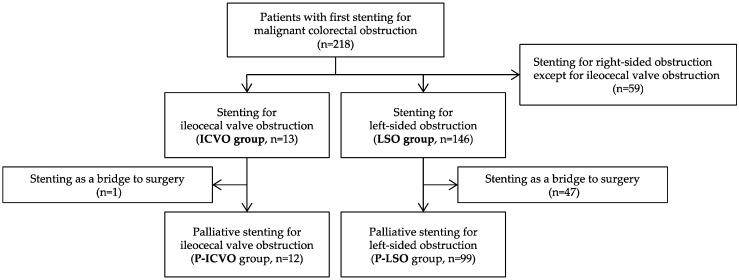
Flowchart of patient selection. ICVO, ileocecal valve obstruction; LSO, left-sided colorectal obstruction.

Between February 2007 and July 2023, 218 consecutive patients with malignant colorectal obstruction who underwent their first SEMS placement were identified using our prospectively maintained database. A total of 159 patients were included in this study, after excluding those with right-sided colonic obstructions other than ICVO (*n* = 59). We classified the patients into two groups, the ileocecal valve obstruction group (ICVO group, *n* = 13) and the left-sided colorectal obstruction group (LSO group, *n* = 146). An LSO was defined as an obstruction located distal to the splenic flexure. Furthermore, patients who underwent SEMS placement for palliative purposes, not as a bridge to surgery, were extracted from the ICVO and LSO groups and designated as the P-ICVO group (*n* = 12) and P-LSO group (*n* = 99), respectively (Figure 1).

### 2.3. SEMS Placement Procedure

All patients received a Niti-S™ colonic stent (Taewoong Medical, Gimpo, Republic of Korea), a WallFlex™ Colonic Stent (Boston Scientific, Marlborough, MA, USA), a JENTLLY Neo Colonic Stent (Japan Life Line, Tokyo, Japan), or a HANAROSTENT Naturfit™ Colon (Boston Scientific, Marlborough, MA, USA), all of which were uncovered SEMSs. The basic details of SEMS placement have been described elsewhere [2,12]. An endoscopic retrograde cholangiopancreatography catheter (ERCP catheter; MTW Co., Ltd., Wesel, Germany) preloaded with a hydrophilic 0.025- or 0.035-inch biliary guidewire (Radifocus, Terumo, Tokyo, Japan; VisiGlide 2, Olympus, Tokyo, Japan) was used to traverse the strictures. In cases where the stricture was strongly bent, Radifocus was used because of its torque transmission and tip flexibility. The guidewire was passed through the stricture using a conventional guidewire technique. After passing the catheter along the guidewire, a contrast agent was injected to assess the stricture under fluoroscopic guidance. If we failed to pass the guidewire through the stricture using the conventional method because of the original acute angulated anatomy or complicated stricture, we used a steerable ERCP catheter (SwingTip, Olympus, TRUEtome, Boston Scientific). After passing through the stricture, we replaced the guidewire with a hard guidewire (Revowave, Piolax Medical Devices, Kanagawa, Japan; Jagwire Plus, Boston Scientific) and inserted the delivery system through the working channel over the guidewire. An SEMS was deployed under fluoroscopic and endoscopic guidance to cover the stricture sufficiently. Colonoscopy and ERCP experts participated in the SEMS placement at our institution. A carbon dioxide insufflation system was used during all endoscopic procedures.

### 2.4. Two-Step Strategy for Colonic Stenting of the ICVO

**Figure 2:**
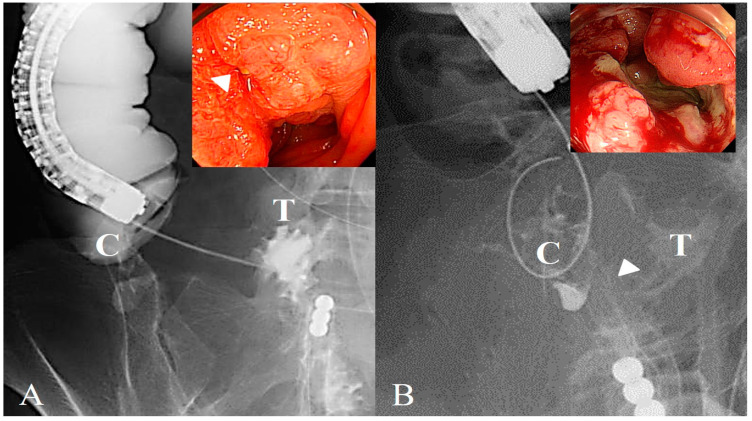
Direct observation of the ileocecal valve obstruction. (**A**) Shows a typical image of a case in which the stricture site is directly observed. The arrowhead in the figure indicates strictures. A guidewire was passed through the stricture using endoscopic and fluoroscopic imaging techniques. (**B**) Shows a typical image of a case in which the stricture site is not directly observed. The arrowhead indicates the estimated connection with the ileocecal junction. A guidewire was passed through the stricture under fluoroscopic guidance. C, cecum; T, terminal ileum.

**Figure 3:**
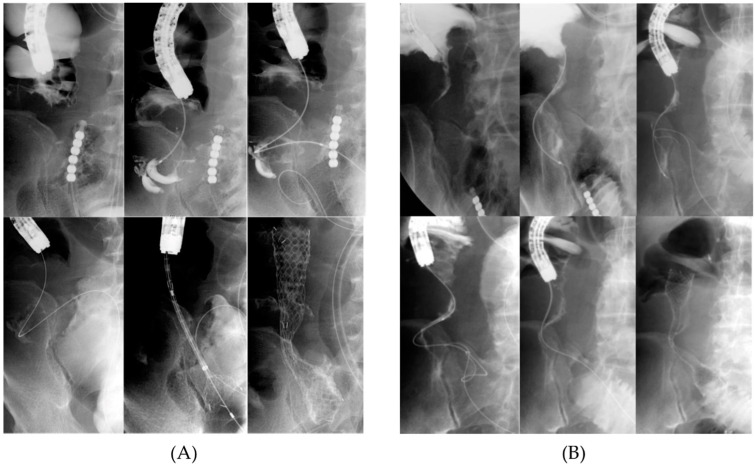
SEMS placement for ileocecal obstruction. (**A**) A contrast agent was injected through an orally placed long intestinal tube to visualize the conjugation and carefully direct a guidewire with ERCP catheter toward the terminal ileum and (**B**) when it was impossible to visualize the conjugation, another guidewire was directed toward the colon through an orally placed long intestinal tube. After successfully identifying the junction using the oral guidewire as a landmark, another guidewire from the anal side was directed towards the terminal ileum for SEMS insertion. ERCP, endoscopic retrograde cholangiopancreatography catheter.

To overcome the challenge of ICVO, we used a strategy involving a two-step procedure for colonic stenting, particularly in cases with severe small-intestine dilation. The first step, inserting a long intestinal tube before colonic stenting, decompressed the dilated small intestine and ensured the safety of the endoscopic procedure. The second step was SEMS placement. If the stricture could not be identified endoscopically (Figure 2) and it was difficult to pass the guidewire through the stricture under endoscopic guidance, contrast media injection or antegrade manipulation through the long intestinal tube facilitated the successful stricture passage of the guidewire (Figure 3).

### 2.5. Outcomes and Definitions

The primary outcome was technical success, and the secondary outcomes included clinical success, recurrent colorectal obstruction (including tumor growth, stent kinking, and food impaction), adverse events (including perforation, bleeding requiring endoscopic hemostasis or transfusion, migration requiring procedure, and tenesmus), time to recurrent colorectal obstruction (TRCRO), and survival time after SEMS placement. The long-term outcomes, TRCRO and survival time after SEMS placement, were evaluated only in the p-ICVO group and p-LSO, group because SEMSs placed as a bridge to surgery were removed during the subsequent surgical intervention.

Technical success was defined as SEMS deployment across the entire length of the stricture on the first attempt with no adverse events. Clinical success was defined as the resolution of symptoms and radiological findings of bowel decompression within 24 h [8].

### 2.6. Statistics

Categorical data are expressed as numbers (%) and were compared using the chi-square test or Fisher’s exact test. Continuous data are expressed as medians (interquartile ranges) and were compared using the Wilcoxon rank-sum test.

TRCRO and survival time were calculated using Kaplan–Meier analysis and compared using the log-rank test. TRCRO was censored when an SEMS was surgically removed along with the tumor, when an SEMS migrated without recurrent colorectal obstruction, when a patient stopped oral intake without recurrent colorectal obstruction, when a patient died, or when a patient was lost to follow-up.

Statistical significance was set at *p* < 0.05. All statistical analyses were conducted using SAS software (version 9.4; SAS Institute, Cary, NC, USA).

## 3. Results

### 3.1. Patient Characteristics

A total of 218 patients underwent their first SEMS placement for colonic obstruction during the study period. After excluding patients with right-sided colonic obstruction other than ICVO (*n* = 59), we identified 13 patients with ICVO (ICVO group) and 146 patients with LSO (LSO group) (Figure 1).

Table 1 shows the patient characteristics of the two groups. Age, sex, primary tumor, the purpose of SEMS placement, and stricture length were not significantly different between the two groups. However, the ICVO group had a significantly lower proportion of patients with a performance status of two or lower (53.9% vs. 80.1%, *p* = 0.039) and a significantly higher rate of long intestinal tube pre-stenting insertion compared to the LSO group (76.9% vs. 6.5%, *p* < 0.001).

Table 2 shows the detailed characteristics of the 13 patients in the ICVO group. The primary tumor was colorectal cancer in ten, bile duct cancer in one, gastric cancer in one, and pancreatic cancer in one. Eleven patients had distant metastases. In seven patients, the orifice of the stricture could not be identified endoscopically. The median stricture length was 5 (3–9) cm.

### 3.2. Procedural and Clinical Outcomes

Table 3 presents the procedural and clinical outcomes of the ICVO group and LSO group. The technical success rates were not significantly different between the ICVO and LSO groups (100% vs. 98.6%, *p* = 1.000). The clinical success rates were also comparable (92.3% vs. 88.4%, *p* = 1.000). The median procedure time was longer in the ICVO group (70 vs. 40 min, *p* < 0.001). While the rates of patients who were able to resume oral intake were not significantly different (100% vs. 93.2%, *p* = 1.000), the median time to resume oral intake was longer in the ICVO group (4 vs. 2 days, *p* = 0.045). The incidence of all adverse events and perforation alone did not differ between the two groups (7.7% vs. 13.0%, *p* = 1.000; 7.7% vs. 6.9%, *p* = 1.000, respectively).

Table 4 shows the details of the procedural and clinical outcomes in the ICVO group. In three patients, an ERCP catheter with controlled steerability was required, and in seven patients, visualization of the stricture through contrast from the long intestinal tube was necessary to pass through the stricture. One patient (No. 10) underwent two SEMS placements to resolve the obstruction, whereas the others underwent one. One patient (No. 4) underwent surgical resection of both the primary tumor and the SEMS 92 days after SEMS placement as a bridge to surgery, while the other patients did not undergo surgery, including ileostomy, during the follow-up period. One patient (No. 8) experienced SEMS migration 29 days after SEMS placement, but no intervention was required. One patient (No. 9) underwent a second SEMS insertion for stent kinking 18 days after the first SEMS insertion, but no further colonic obstruction occurred until death. One patient (No. 10) underwent a second SEMS insertion for re-obstruction due to mucosal hyperplasia 7 days after the initial placement of two SEMSs. Additionally, after approximately 8 months of chemotherapy, a skin fistula developed at the site of the second SEMS. Fortunately, that SEMS appeared to have been ejected through the anus, and he recovered by using the fistula like a colostomy without surgery. The patient did not require further intervention until death. One patient (No. 13) did not achieve clinical success, but bowel decompression was observed and oral intake was resumed 4 days after SEMS placement.

### 3.3. TRCRO and Survival Time of Patients with Palliative SEMS Placement

**Figure 4:**
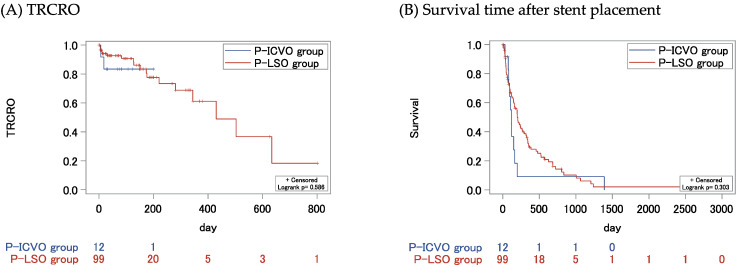
Kaplan–Meier estimates of (**A**) TRCRO and (**B**) survival time after SEMS placement of the P-ICVO group and P-LSO group. TRCRO, time to recurrent colorectal obstruction; ICVO, ileocecal valve obstruction; LSO, left-sided colorectal obstruction.

Figure 4 shows the TRCRO scores and survival rates after SEMS placement in the P-ICVO group and P-LSO group. The median TRCRO and survival time were not significantly different between the two groups (not reached vs. 430 days, *p* = 0.586; 119 days vs. 200 days, *p* = 0.303). Table S1 presents the characteristics and outcomes of the P-ICVO group and P-LSO group. The rates of patients who received chemotherapy after SEMS placement and the median time to start chemotherapy did not differ between the two groups (50.0% vs. 50.5%, *p* = 0.974; 9.5 days vs. 10 days, *p* = 0.652, respectively).

## 4. Discussion

In this study, we investigated SEMS placement across different malignant ICVO cases and compared its clinical outcomes with those of SEMS placement for malignant LSO. The technical (100% vs. 98.6%, *p* = 1.000) and clinical success rates (92.3% vs. 88.4%, *p* = 1.000) were comparable between the two groups. The incidence of all adverse events and perforation alone in the two groups were also comparable (7.7% vs. 13.0%, *p* = 1.000; 7.7% vs. 6.9%, *p* = 1.000, respectively). When comparing the P-ICVO group and P-LSO group, both of which underwent palliative SEMS placement, the TRCRO (not reached vs. 430 days, *p* = 0.586) and survival time after SEMS placement (119 days vs. 200 days, *p* = 0.303) were not significantly different. These results suggest that SEMS placement for ICVO is as safe and effective as that for LSO, which is an already established procedure. To the best of our knowledge, this is the largest reported case series of SEMS placement for malignant ICVO.

SEMS insertion for right-sided obstructions has generally been reported to have lower success rates than that for left-sided obstructions [13]. This has been attributed to the technical difficulties encountered in placement on the right side due to the long distance from the anus, poor preparation, and tortuosity of the bowel [14,15]. For ICVO, the original angulated anatomy makes SEMS placement more difficult [10]. In fact, of the 13 cases in our ICVO group, the stricture could not be directly viewed in 7 cases, and an ERCP catheter with controlled steerability was required in 3 cases. Additionally, the procedure time was longer in the ICVO group than that in the LSO group. As previously reported, stenting for ICVO is difficult; however, the technical success rate of the ICVO group was comparable to that of the LSO group in this study.

There are two possible reasons as to why similar technical and clinical success rates were achieved despite these difficulties. First, a Two-Step Strategy was used in 10 of the 13 cases. Decompression of the oral intestinal tract in advance allows for stenting to be performed in a good general condition. In addition, when not directly visible, contrasting the oral side was helpful for identifying the stricture site and passing the guidewire. In their case series, Ishii et al. reported the usefulness of tube placement in the long intestine before SEMS placement for the treatment of malignant ileocecal obstruction [10]. Second, both colonoscopy and ERCP experts participated in SEMS placement at our institution. To insert an endoscope from the anus to the ileum in a poorly pretreated bowel, the surgeon must be proficient in colonoscopy. Guidewire manipulation only under fluoroscopic guidance in cases where the stricture site cannot be directly viewed and the use of a catheter with controlled steerability are techniques often used in ERCP. In a previous report, fewer complications of colorectal stenting were associated with surgeons familiar with ERCP [11].

In the present study, adverse events in the ICVO group occurred in 7.7% (one patient) of the cases, which was comparable to the incidence in the LSO group. The adverse event in this patient was a skin fistula, but it did not require surgical intervention. The characteristics of the Niti-S stent, the Naturfit stent, and the JENTLLY stent, which were used in the ICVO group, might have contributed to this safety. These SEMSs are characterized by a low axial force and an ‘AF zero border’ of 30 degrees or more [16,17,18]. Axial force is defined as the recovery force when the SEMS is bent [19], and the AF zero border is defined as the angle at which the axial force disappears when the SEMS is stretched. An SEMS with a high axial force and without an AF zero border may cause gastrointestinal perforation owing to the pressure load at the SEMS’s edges. A low axial force and a large AF zero border may be particularly effective for tortuous portions of the digestive tract such as the ileocecal region [16].

SEMS placement for ICVO may also have potential applications in benign conditions, particularly in Crohn’s disease. The ileocolonic region is the most common site of both primary and anastomotic strictures in Crohn’s disease [20], and for such strictures, the temporary placement of a fully covered SEMS could be considered as a therapeutic option, especially in cases where endoscopic balloon dilation or endoscopic electroincision have failed [21]. Additionally, a case report described successful SEMS placement as a bridge to surgery for acute bowel obstruction due to a stricture at ileocolonic anastomosis in a Crohn’s disease patient [22]. Given that the high technical success rate in our ICVO group was achieved using the Two-Step Strategy, similar technical success might be expected for Crohn’s disease-related ICVO using the same approach, although further studies are needed to validate this approach in benign conditions.

This study has several limitations. First, it is a single-center retrospective study and includes only a small number of cases of SEMS placement for ICVO. Therefore, statistical equivalence could not be demonstrated in the comparison with cases of SEMS placement for LSO. Second, we did not compare cases of ICVO treated with SEMS placement to those treated with surgery, which represents the true clinical question. However, a retrospective report showed high mortality (9–14.5%) and morbidity (32–54.3%) rates after emergency surgery with colectomy for obstructive right-sided colorectal cancer [23]. In addition, ileostomy, which is sometimes performed for ICVO, has higher occurrence rates of early and late adverse events than colostomy [24]. Considering the invasiveness of surgery and the reduced quality of life caused by ileostomy, SEMS placement may be a useful option for ICVO. Prospective studies comparing SEMS placement and surgery are required.

In conclusion, endoscopic SEMS placement for malignant ICVO is as safe and effective as that for malignant LSO. The Two-Step Strategy might be useful for overcoming its technical challenges.

## Figures and Tables

**Table 1:** Patient characteristics of the ICVO group and LSO group.

Variable	ICVO Group (*n* = 13)	LSO Group (*n* = 146)	*p*-Value
Age (year)	67 (62–79)	69 (60–78)	0.833
Male sex	8 (61.5)	80 (54.8)	0.639
PS * ≤ 2	7 (53.9)	117 (80.1)	0.039
Primary tumor			0.955
Colorectal cancer	10 (76.9)	94 (64.4)	
Gastric cancer	1 (7.7)	23 (15.8)	
Pancreatic cancer	1 (7.7)	13 (8.9)	
Other tumors ^†^	1 (7.7)	16 (11.0)	
Long intestinal tube insertion	10 (76.9)	10 (6.9)	<0.001
Purpose of SEMS placement			0.111
Bridge to surgery, *n* (%)	1 (7.7)	47 (32.2)	
Palliation, *n* (%)	12 (92.3)	99 (67.8)	
Stricture length (cm)	5 (3–9)	4 (3–6)	0.455

All values are expressed as n (%) or median (interquartile range). * Eastern Cooperative Oncology Group performance status. ^†^ Other tumors include gynecological cancer (*n* = 8), urinary tract cancer (*n* = 3), biliary tract cancer (*n* = 4), adenoid cystic carcinoma of the soft palate (*n* = 1), and liposarcoma of the iliopsoas muscle (*n* = 1). ICVO, ileocecal valve obstruction; LSO, left-sided colorectal obstruction.

**Table 2:** Detailed characteristics of patients in the ICVO group.

Patient	Age, y/Sex	PS *	Primary Tumor	Metastatic Sites	Major Comorbidity	Long Intestinal Tube Insertion	Stricture Visibility	Purpose of Stenting	Stricture Length, cm
1	79/F	2	Colorectal cancer	Liver, lung, and pancreas	Ischemic heart disease	Yes	No	Palliation	5
2	86/M	4	Colorectal cancer	None	Atrial fibrillation and cerebral infarction	Yes	Yes	Palliation	3
3	65/M	3	Colorectal cancer	Peritoneum and bone	None	Yes	No	Palliation	9
4	64/M	1	Colorectal cancer	Para-aortic lymph nodes and liver	Ischemic heart disease and diabetes mellitus	Yes	No	BTS ^†^	7
5	84/F	3	Colorectal cancer	Liver, lung, and peritoneum	Bronchial asthma	Yes	No	Palliation	3
6	50/M	2	Bile duct cancer	Peritoneum and bone	None	Yes	No	Palliation	9
7	71/F	2	Colorectal cancer	Liver and lung	None	Yes	No	Palliation	4
8	60/F	3	Colorectal cancer	Liver	None	No	Yes	Palliation	1
9	62/M	3	Colorectal cancer	Liver, lung, and peritoneum	None	Yes	Yes	Palliation	5
10	42/M	1	Gastric cancer	Peritoneum	None	Yes	No	Palliation	7
11	89/F	4	Colorectal cancer	None	Transient ischemic attack, dementia, and dysphagia	Yes	Yes	Palliation	3
12	67/M	1	Colorectal cancer	Peritoneum and bone	None	No	Yes	Palliation	12
13	76/M	1	Pancreatic cancer	Liver and peritoneum	Diabetes mellitus	No	Yes	Palliation	3

* Eastern Cooperative Oncology Group performance status. ^†^ Bridge to surgery. ICVO, ileocecal valve obstruction.

**Table 3:** Procedural and clinical outcomes of the ICVO group and LSO group.

Variable	ICVO Group (*n* = 13)	LSO Group (*n* = 146)	*p*-Value
Type of SEMS			
Niti-S, 20 mm, 80 mm	0 (0)	7 (4.8)	
Niti-S, 20 mm, 100 mm	2 (15.4)	9 (6.2)	
Niti-S, 20 mm, 120 mm	4 (30.8)	3 (2.1)	
Niti-S, 22 mm, 60 mm	0 (0)	1 (0.7)	
Niti-S, 22 mm, 80 mm	1 (7.7)	16 (11.0)	
Niti-S, 22 mm, 100 mm	0 (0)	14 (9.6)	
Niti-S, 22 mm, 120 mm	3 (23.1)	28 (19.2)	
WallFlex, 22 mm, 60 mm	0 (0)	15 (10.3)	
WallFlex, 22 mm, 90 mm	0 (0)	5 (3.4)	
WallFlex, 22 mm, 120 mm	0 (0)	2 (1.4)	
JENTLLY 22 mm, 60 mm	0 (0)	1 (0.7)	
JENTLLY 22 mm, 80 mm	0 (0)	6 (4.1)	
JENTLLY 22 mm, 100 mm	0 (0)	6 (4.1)	
JENTLLY 22 mm, 120 mm	1 (7.7)	9 (6.2)	
Naturfit 22 mm, 60 mm	0 (0)	1 (0.7)	
Naturfit 22 mm, 90 mm	0 (0)	7 (4.8)	
Naturfit 22 mm, 120 mm	1 (7.7)	8 (5.5)	
Multiple stenting	1 (7.7)	8 (5.5)	
Procedure time (min)	70 (55–84)	40 (30–55)	<0.001
Technical success	13 (100)	144 (98.6)	1.000
Clinical success	12 (92.3)	129 (88.4)	1.000
Recurrent colorectal obstruction	2 (15.4)	18 (12.3)	0.669
Tumor growth	1 (7.7)	12 (8.2)	1.000
Stent kinking	1 (7.7)	2 (1.4)	0.227
Stool impaction	0 (0)	4 (2.7)	1.000
Adverse events			
All adverse events	1 (7.7)	19 (13.0)	1.000
Perforation	1 (7.7)	10 (6.9)	1.000
Bleeding	0 (0)	1 (0.7)	1.000
Migration requiring procedure	0 (0)	2 (1.4)	1.000
Tenesmus	0 (0)	7 (4.8)	1.000
Oral intake after SEMS placement	13 (100)	136 (93.2)	1.000
Time to resume oral intake, days	4 (3–4)	2 (2–3)	0.045

All values are expressed as *n* (%) or median (interquartile range). ICVO, ileocecal valve obstruction; LSO, left-sided colorectal obstruction.

**Table 4:** Details of procedural and clinical outcomes in the ICVO group.

Patient	Use of a Steerable ERCP Catheter	Contrast of the Stricture from the Long Intestinal Tube	Type of SEMS	Procedure Time, min	Clinical Success	Technical Success	Recurrent Colorectal Obstruction	Adverse Events	Treatment After Stenting	TRCRO, Days	Survival Time, Days
1	No	Yes	Niti-S, uncovered, 20 mm, 100 mm	102	Yes	Yes	No	No	BSC	200 *	200
2	No	No	Niti-S, covered, 20 mm, 120 mm	67	Yes	Yes	No	No	BSC	163 *	163
3	No	Yes	Niti-S, uncovered, 20 mm, 120 mm	84	Yes	Yes	No	No	Chemotherapy	107 *	107
4	No	Yes	Niti-S, uncovered, 20 mm, 100 mm	46	Yes	Yes	No	No	Operation	92 ^†^	1639
5	No	No	Niti-S, uncovered, 22 mm, 120 mm	72	Yes	Yes	No	No	BSC	83 *	83
6	No	Yes	Niti-S, uncovered, 22 mm, 120 mm	75	Yes	Yes	No	No	Chemotherapy	75 *	75
7	Yes	No	Niti-S, uncovered, 20 mm, 120 mm	70	Yes	Yes	No	No	BSC	31 *	31
8	No	Yes	Niti-S, uncovered, 20 mm, 120 mm	55	Yes	Yes	No	No	Chemotherapy	29 ^‡^	119
9	No	No	Niti-S, uncovered, 22 mm, 120 mm	31	Yes	Yes	Stent kinking	No	Chemotherapy	18	94
10	Yes	Yes	Niti-S, uncovered, 22 mm, 100 mm and Niti-S, uncovered, 22 mm, 120 mm	120	Yes	Yes	Tumor ingrowth	Perforation (Skin fistula)	Chemotherapy	7	1390
11	Yes	Yes	JENTLLY, uncovered, 22 mm, 120 mm	163	Yes	Yes	No	No	BSC	122 *	122
12	No	No	Naturfit, uncovered, 22 mm, 120 mm	70	Yes	Yes	No	No	Chemotherapy	69 ^§^	69 (Alive) ^§^
13	No	No	Niti-S, uncovered, 22 mm, 80 mm	35	No	Yes	No	No	BSC	149 *	149

* The patients had patent SEMSs when they died. ^†^ The patient had surgical resection of the primary tumor including the SEMS, 92 days after SEMS placement. ^‡^ The SEMS was migrated 29 days after SEMS placement, but no intervention was required. ^§^ The patient was alive and had the SEMS patent at the time of lost to follow-up 149 days after SEMS placement. ICVO, ileocecal valve obstruction; TRCRO, time to recurrent colorectal obstruction; BSC, best supportive care.

## Data Availability

The original contributions presented in this study are included in the article/Supplementary Materials. Further inquiries can be directed to the corresponding author.

## Supplementary Materials

The following supporting information can be downloaded from https://www.mdpi.com/article/10.3390/jcm14030826/s1. Table S1: Characteristics and outcomes of the P-ICVO group and P-LSO group.

## Abbreviations

The following abbreviations are used in this manuscript:

SEMS	Self-expandable metallic stent
LSO	Left-sided colorectal obstruction
ICVO	Ileocecal valve obstruction
ERCP	Endoscopic retrograde cholangiopancreatography
TRCRO	Time to recurrent colorectal obstruction
BTS	Bridge to surgery
BSC	Best supportive care
