# Supplementary material for: Safety and Effectiveness of Colonic Stenting for Ileocecal Valve Obstruction and Usefulness of Two-Step Strategy: Single-Center Retrospective Study
Source: J Clin Med. 2025 Jan 27;14(3):826. doi: 10.3390/jcm14030826 (PMC11818438; doi:10.3390/jcm14030826)
Supplement: Supplementary file 1 [file jcm-14-00826-s001.zip › jcm-3440651-supplementary.pdf]

**Table S1.** Characteristics and outcomes of the P-ICVO group and P-LSO group.

| Variable | ICVO group (n =12) | LSO group (n = 99) | P-value |
|---|---|---|---|
| Age (year) | 69 (61–81.5) | 67 (56–78) | 0.488 |
| Male Sex | 7 (58.3) | 54 (54.6) | 0.803 |
| PS* ≤ 2 | 6 (50.0) | 79 (79.8) | 0.032 |
| Primary caner | | | 0.456 |
| Colorectal cancer | 9 (75.0) | 47 (47.5) | |
| Gastric cancer | 1 (8.3) | 23 (23.2) | |
| Pancreatic cancer | 1 (8.3) | 13 (13.1) | |
| Other cancers† | 1 (8.3) | 16 (16.2) | |
| Pre-stenting insertion of the long intestinal tube | 9 (75.0) | 9 (9.1) | <0.001 |
| Stricture length (cm) | 4.5 (3–9) | 4 (3–7) | 0.862 |
| Clinical success | 11 (91.7) | 83 (83.8) | 0.688 |
| Technical success | 12 (100.0) | 97 (98.0) | 1.000 |
| Recurrent colorectal obstruction | 2 (16.7) | 18 (18.2) | 1.000 |
| Tumor growth | 1 (8.3) | 12 (12.1) | 1.000 |
| Stent kinking | 1 (8.3) | 2 (2.0) | 0.293 |
| Stool impaction | 0 (0.0) | 4 (4.0) | 1.000 |
| Adverse events | | | |
| All adverse events | 1 (8.3) | 17 (17.2) | 0.687 |
| Perforation | 1 (8.3) | 10 (10.1) | 1.000 |
| Bleeding | 0 (0.0) | 1 (1.0) | 1.000 |
| Migration requiring procedure | 0 (0.0) | 1 (1.0) | 1.000 |
| Tenesmus | 0 (0.0) | 6 (6.1) | 1.000 |
| Oral intake after stent placement | 12 (100.0) | 89 (89.9) | 0.597 |
| Time to resume oral intake, days | 4 (3–4.5) | 2 (2–3) | 0.009 |
| Chemotherapy after stent placement | 6 (50.0) | 50 (50.5) | 0.974 |
| Time to start chemotherapy, days | 9.5 (8–19) | 10 (6–21) | 0.652 |

All values are expressed as n (%) or median (interquartile range).
* Eastern Cooperative Oncology Group performance status.
ICVO, ileocecal valve obstruction; LSO, left-sided colorectal obstruction.
